# Bacterial Biofilms—A Threat to Biliary Stents, Understanding Their Formation, Clinical Consequences and Management

**DOI:** 10.3390/medicina61030512

**Published:** 2025-03-16

**Authors:** Jolanta Gruszecka, Rafał Filip

**Affiliations:** 1 Institute of Health Sciences, Medical College of Rzeszow University, 35-959 Rzeszow, Poland; 2 Department of Clinical Microbiology, Clinical Hospital No. 2, 35-301 Rzeszow, Poland; 3 Faculty of Medicine, University of Rzeszow, 35-959 Rzeszow, Poland; 4 Department of Gastroenterology with IBD Unit, Clinical Hospital No. 2, 35-301 Rzeszow, Poland

**Keywords:** biofilm, biliary tract stents, biofilm elimination

## Abstract

A biofilm is a community of microbial cells which are enclosed in an external matrix and separated by a network of water channels attached to natural or artificial surfaces. Biofilms formed inside biliary stents consist of a mixed spectrum of bacterial communities, most of which usually originate from the intestines. The patency of biliary stents is the most important problem. Stent occlusion can threaten the health and even life of patients. The main cause of this phenomenon is bile sludge, which is an excellent environment for the multiplication and existence of microorganisms. Due to the great clinical importance of maintaining the patency of biliary stents, several methods have been developed to prevent the accumulation of sludge and the subsequent formation of biofilm; these include, among others, the use of anti-adhesive materials, coating the inner surface of stents with metal cations (silver, copper) or other antimicrobial substances, the implementation of biodegradable drug-eluting biliary stents and the development of a new stent design with an anti-reflux effect. This article presents the latest information on the formation of biofilms in biliary stents, as well as historical and future methods of prevention.

## 1. Introduction

In the 1940s, it was observed that the majority of microorganisms in the aquatic environment formed aggregates that adhered to objects immersed in water, exhibiting different properties from microorganisms that occur as single cells [1]. This specific form of existence of bacteria and fungi was called biofilm [2]. It has an advantage over planktonic forms (occurring as single, scattered cells, most often in an aquatic environment) in that it provides a greater chance of survival in a changing environment [3,4,5,6]. An important feature of a biofilm is its reduced sensitivity to physicochemical factors as well as stress [7]. In their natural habitat, more than 90% of bacteria occur in this form [3,8]. Biofilms are communities of microorganisms that adhere to each other and are embedded in an extracellular matrix with a diverse chemical and structural composition created by the microorganisms themselves [9,10]. The gradients that exist in the biofilm matrix allow for the formation of microniches, created by different microorganisms [9]. Anaerobic microorganisms and cells which are more sensitive to environmental stressors, such as hazardous chemicals, inappropriate pH, or physical damage, can live in deeper layers of biofilm [11]. The top layers of the biofilm, with an appropriate partial oxygen concentration and access to nutrients, enable microbial cells to carry out active metabolic processes with a high rate of division [12].

The goal of this review thesis was to present the issues related to the phenomenon of biofilm formation on medical devices, especially in biliary tract stents, as well as to present the methods of preventing and combating this very dangerous process for patients.

## 2. Biofilm Formation

**Figure 1:**
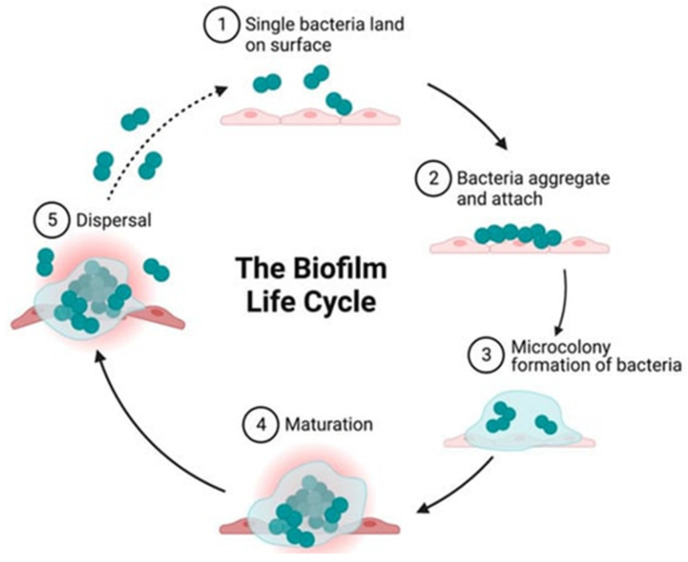
Stages of biofilm creation [14].

Biofilm formation is a complex process. The biofilm life cycle consists of distinct stages: (1) initialization, (2) bacterial adhesion and aggregation, (3) microenvironment formation, (4) microenvironment maturation, (5) dispersion and (6) quorum sensing QS, as shown in Figure 1 [13,14,15].

The first reversible stage of initialization occurs mainly due to physicochemical reactions between the colonized natural or artificial surface and the microbial cell [16]. Reversible binding most often occurs as a result of electrostatic, hydrophobic, van der Waals and surface tension interactions, or due to gravitational forces [17]. During the reversible attachment stage, microbial cells come into contact with the surface and begin to adhere, but can still be relatively easily removed. Reversible binding is generally mediated by the proteins found on the surface of the microorganisms. The rate of microbial adhesion is significantly dependent on the characteristics of the colonized surface, including hydrophobicity, topography and charge [18]. The chemical composition of the pathogen’s cell wall and the roughness of the substrate surface are extremely important at this stage, because both factors affect the type of physicochemical interactions [7].

During the adhesion and aggregation phase of bacteria, irreversible attachment occurs; the cells completely bind to the surface and begin to produce an extracellular matrix that prevents their physical removal from the surface [2,13]. After attachment, the microorganisms change their profile from planktonic to sessile. The composition of the biofilm is different; it is a mixture of various secreted biomolecules: polysaccharides, proteins, lipids, teichoic acids and environmental DNA (eDNA) [11].

In the phase of microenvironment formation, biofilms grow and gain a three-dimensional structure due to cell proliferation, adhesion between microbial cells and the secretion of extracellular mucus [19]. Cells in the center of the biofilm, which have limited access to oxygen and nutrients, can often become dormant. These microorganisms are metabolically dormant, but not dead [20,21]. Anaerobic metabolic pathways become dominant among the microorganisms living deep in the biofilm due to their limited access to oxygen and nutrients [17,19,22].

During the maturation stage, changes and differentiation occur among the cells of the microorganisms forming the biofilm [7,16]. Differences in the metabolism of microorganisms can be observed depending on their location in the biofilm structures [23]. In the biofilm, there is increased diversity in activities of the cells forming it. There are dead cells, dormant cells and cells with aerobic and anaerobic metabolism [2].

In the dispersion stage, the mature biofilm reaches a critical size, bursts and disperses planktonic microorganisms [13]. Active detachment is triggered by various environmental signals such as changes in temperature, pH, nitric oxide, nutrient deficiency, oxygen deficiency and other stress factors [24,25]. The resulting chemical gradients experienced by the cells in the biofilm are believed to be the main causes of its dispersion [26].

An important factor in biofilm structures is the phenomenon of quorum sensing (QS). It represents chemical communication through signal substances or autoinducers (farnesol, tyrosol, dodecanol) which accumulate with increasing cell density, responding to changes in the external environment as well as to processes inside the biofilm. Microorganisms use autoinducers to regulate the course of physiological processes or the expression of pathogenicity factors in a controlled manner, depending on their number [27]. When the appropriate number of cells, i.e., the quorum, is reached, the concentration of the autoinducer exceeds the threshold value, and the controlled regulation of gene expression occurs, which enables the cooperation of a given population of microorganisms and may cause the simultaneous production of virulence factors. These factors affect, among others, sensitivity or resistance to biocides [2,23,28,29]. The metabolic diversity of microorganisms in individual layers of the biofilm may also lead to a differing sensitivity to antibiotics [12]. The QS system occurs both between cells of the same and different species and provides an opportunity for the coordinated regulation of important life processes in the entire population [2].

## 3. Biofilm Distribution

Biofilms are ubiquitous in almost every environment, affecting human health and industry [3,30]. They have been created on a variety of surfaces in different habitats, both natural and man-made, including in the hospital environment [30,31]. One of the first to recognize the importance of biofilms in medicine was Niels Høiby [32]. Since then, this phenomenon has been supported by numerous pieces of evidence [32]. Biofilms are involved in many different bacterial infections in the body. The National Institutes of Health (NIH) revealed that of all bacterial infections, 60–80% are associated with biofilm formation [15,33]. Biofilms are formed on various medical devices such as contact lenses, catheters, prostheses, biliary stents, valves and pacemakers, but also on various surfaces of the human body, including the skin or the mucous membranes of the respiratory and digestive tracts, constituting an important reservoir for the initiation of new infections [11]. Environmental biofilms in drinking water systems may be a source of the respiratory pathogen *Legionella pneumophila*, the causative factor of Legionnaires’ disease, and opportunistic pathogens such as *Mycobacterium avium*, which poses a health risk, especially to immunocompromised patients. *Legionella* spp. often form biofilms, particularly in shower houses, which are believed to promote the persistence and resistance of the respiratory pathogen to chlorine [34]. Cholera, a waterborne diarrheal disease, is caused by *Vibrio cholerae*. This pathogen moves between the water body, where it forms biofilms on chitinous surfaces, and the human body, where it successfully colonizes the gastrointestinal tract. Studies with neonatal mice showed that both intact biofilms and dispersed sessile *V. cholerae* cells are more infectious than free-living planktonic cells [35]. From a clinical point of view, the most important features of a biofilm are its high resistance to antimicrobial agents and the immune system, as well as its strong ability to colonize patient tissues and biomedical materials [36]. Studies have shown that bacteria originating from biofilms are characterized by a higher resistance to antimicrobial compounds than their individual, planktonic counterparts [13]. Factors that cause higher antibiotic resistance in biofilm-associated infections include the following: metabolic changes in bacterial cells, antibiotic inactivation and reduced penetration through the extracellular matrix, inoculum effects related to the high density of bacterial cells in relation to the number of available antibiotic molecules and the increased exchange of resistance mechanisms between bacteria in close proximity to each other [11]. Biofilm-forming microorganisms may be dangerous for patients with predisposing factors, such as comorbidities or immunosuppression.

## 4. Biofilms in the Human Body and on Medical Equipment and Devices

Biofilms form on biomaterials, such as dental prostheses, catheters, endoprostheses, biliary tract stents, as well as on living tissues. Microorganisms within the biofilms are up to 1000 times more tolerant to antibiotic therapy than their planktonic counterparts, which allows them to evade elimination excellently [13]. Opportunistic biofilms readily colonize virtually any surface, especially those that are foreign to the body, such as implanted medical devices, used both in the short-term and for extended periods of time. As various medical devices are increasingly used in all branches of medicine, strategies to control biofilm formation in various environments are of great importance [37]. The spread of biofilms on medical implants is one of the main factors triggering persistent and chronic infections in clinical settings [38]. Biofilm formation and microbial colonization are encountered on a wide variety of implantable medical devices. Common examples include catheters, feeding tubes, cochlear implants, cardiac valves and pacemakers, urologic and breast implants, biliary stents, endoscopic tubes, contact lenses and neurosurgical and orthopedic implants. The abundance of microorganisms on various surfaces and sites in the body is observed depending on environmental characteristics, such as the presence of fluid flow and the surface properties of the implants, as well as the interplay between colonization and the human immune response [38]. The extracellular matrix protects microbial cells from drying out, constituting a barrier that impedes the penetration of antibiotics and antiseptics, impedes the interaction of the host’s immune system (including impeding phagocytosis and inhibiting the penetration of antibodies), reduces the effective concentration of antibiotics reaching bacterial cells and creates optimal conditions for the formation of microbial colonies [7,9].

Multi-species biofilms in the human body can be both a positive and negative phenomenon. They are created by microbiota living in the oral cavity—mainly on the surface of teeth, in the intestines, in the vagina or on the skin [2]. There are over 700 different species of bacteria in the human oral cavity. They can initiate the formation of dental biofilms, also known as dental plaque. The exact composition of dental biofilms varies both from site to site in the mouth and from person to person. The core composition of the microbiome has been proposed to include species from the following genera: *Streptococcus*, *Veillonella*, *Granulicatella*, *Neisseria*, *Haemophilus*, *Corynebacterium*, *Rothia*, *Actinomyces*, *Prevotella*, *Capnocytophaga*, *Porphyromonas* and *Fusobacterium* [39]. Dental biofilm is a permanent reservoir of microorganisms, which can systematically spread throughout the body. Dental biofilm bacteria are also directly and indirectly associated with various systemic diseases, such as aspiration pneumonia, premature birth and low-birth-weight children, diabetes, circulatory system diseases, atherosclerosis and infective endocarditis [40]. For example, the caries production of *Streptococcus mutans* results from the adhesive properties (biofilm) of the extracellular polymeric substances secreted by it, the production of which is partially stimulated by the presence of fructose and the conversion of simple sugars into intracellular polysaccharides (mutan, dextran, levan) [41,42]. The final bacterial metabolites that make up dental plaque are organic acids that damage the enamel, allowing various cariogenic bacteria to begin the process of tooth destruction [42]. *S. mutans* can also cause bacterial endocarditis, especially the subacute clinical form in 50–70% of all cases of this disease entity. In people with risk factors for the development of the disease, which include congenital heart defects, rheumatic fever, heart surgery and damage to the oral mucosa, streptococci are allowed to enter the blood vessels. This can cause transient bacteremia with heart valve colonization and biofilm formation [42]. The intestinal biofilm, built by multi-species microorganisms, protects against chronic gastrointestinal diseases, retains water in the body, stimulates the host’s immunity and participates in the production of vitamins (vitamin K, biotin) and the breakdown of food. The gastrointestinal microflora contains more than 1000 microbial species and the intestinal biofilm is formed by, among others, bacteria of the genera *Bacteroides*, *Bifidobacterium*, *Enterococcus* and *Streptococcus* [43]. The ability to form biofilms is also characteristic of the lactic acid bacteria of the *Lactobacillus* genus. Colonizing the vagina and intestines, they protect against infections of the digestive tract, urinary tract and sexually transmitted diseases. In the vagina, these bacteria participate in the protection of the mucous membrane against pathogens, secreting metabolites (organic acids, hydrogen peroxide, bacteriocins) with antimicrobial activity [44,45]. Over 60% of the microorganisms colonizing human skin are various bacteria that form biofilms. The dominant flora includes *Staphylococcus* spp., *Corynebacterium* spp. and *Propionibacterium* spp. [46]. The natural microflora on the surface of healthy skin performs a protective function; a biofilm is the predominant form of microbial life on its surface [2].

Modern medicine increasingly relies on surgical interventions and the placement of permanent medical devices in the patient’s body. Both surgical procedures and medical devices can introduce foreign microorganisms into the body, which can serve as a permanent reservoir of infection and cause biofilm formation. Almost 80% of device-related infections are caused by biofilms formed by Gram-positive *Staphylococcus* spp. bacteria, primarily *Staphylococcus epidermidis* and *Staphylococcus aureus* [47]. *Staphylococcus* spp. are a commensal of the skin, but in favorable conditions they can cause infection. They can be introduced into the body via contaminated medical devices, from medical personnel or from patients themselves [21]. Medical devices are made of many materials, including metals, plastics and ceramics. Plastics are more easily colonized than metal surfaces, but bacterial biofilms can form on both surfaces [48]. The van der Waals and hydrophobic forces are the main factors influencing the adhesion of bacteria to the surfaces of medical devices [49]. Surface characteristics, including hydrophobicity, texture and electrostatic charge, can facilitate the attachment of microorganisms and influence which strains have an affinity for it [13]. The most frequently isolated bacterial strains associated with biofilms in medical devices used on the long-term and short-term are presented in Table 1.

## 5. Biofilms on the Inner Surface of Biliary Stents

Plastic stents in the biliary tract are often occluded by biliary sludge, which provides an excellent environment for microorganisms to adhere, multiply and thrive in. This is an additional factor contributing to biliary stent obstruction [16]. Stent patency is a major concern for patients, endoscopists and physicians, because it can affect both the life expectancy and treatment schedule of patients and depends on biliary tract injury and stent location. Biliary stent occlusion can occur due to several factors: biliary sludge causing the slowing of bile flow, bile viscosity, food exposure and the subsequent formation of a coating from dietary fibers. The reflux of intestinal contents into the bile duct allows for the easy adhesion, colonization and growth of bacteria on the inner surface of the stent, leading to an ascending bacterial infection. No ideal stent with permanent patency has been identified to date [62,63,64].

**Figure 2:**
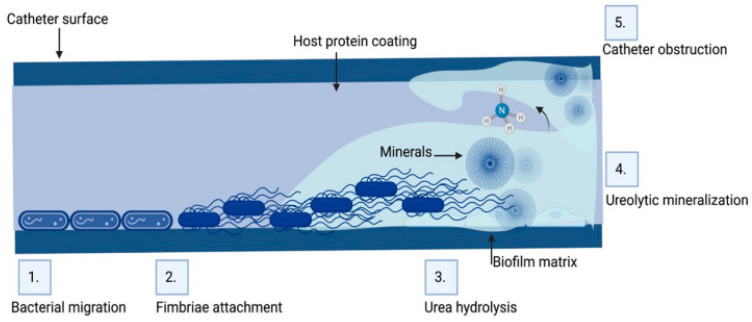
Biofilm formation on the inner surface of biliary tract stents [14].

Microorganisms isolated from obstructed biliary stents (anaerobic and aerobic bacteria and fungi) secrete several types of proteins, such as fibronectin, vitronectin, laminin, fibrin and collagen, which increase their adhesion. It is believed that the biofilm on the inner surface of the stent causes it to be irregular, which further facilitates the accumulation of sediment and debris, precipitating the occurrence of obstruction and the recurrence of cholangitis [65]. Biofilms formed inside stents consist of a mixed spectrum of microorganisms [10]. Polymicrobial communities act synergistically on biofilm maturation, causing it to gradually become thicker [66]. Stent occlusion leads to jaundice and bacterial cholangitis with polymicrobial infections in 90% of patients, as shown in Figure 2 [16]. The inappropriate use of antimicrobial agents may lead to the emergence of antimicrobial resistance and, consequently, ineffective treatment of stent-related cholangitis [67].

Currently, more than 70% of patients with biliary jaundice are treated with the implantation of a biliary stent made of plastic or metal [68]. Plastic stents can be removed and replaced if necessary, which is their main advantage. Self-expanding metal stents are durable and have the advantages of a larger lumen and a longer period of patency [68]. In recent years, biodegradable biliary stents have also been developed for endoscopic applications [69]. Studies comparing the properties and safety of different types of stents for preoperative biliary drainage are limited, and no consensus has yet been reached on the optimal type [70].

A study conducted in Italy analyzed the composition of biofilms colonizing biliary stents. For this purpose, biliary stents were collected from 56 patients. The study participants were 32 to 89 years old (mean 67.30 ± 15.75) and had been wearing stents for 13 to 330 days (mean 70.21 ± 73.35). All stents were collected from patients who had not undergone antibiotic prophylaxis or chemotherapy. The time of stent patency ranged from 5 to 330 days [68]. The study used metal stents made of a braided nickel–titanium alloy (Nitinol) with a full-length silicone polymer lining, or plastic—made of polyethylene. The species associated with stents were usually anaerobic and Gram-positive bacteria, comprising 50% and 58.3%, respectively. The three species *Streptococcus anginosus*, *Escherichia coli* and *Enterococcus faecalis* were found in more than 80% of the samples (prevalence = 83.0%) [68].

In another study, a prospective microbiological analysis of biliary stent biofilm from all patients requiring elective or emergency stent replacement/removal was performed in northern India between April 2011 and March 2014. A total of 81 patients (41 males) aged 20–86 years were included in the study. The primary reasons for stent placement were gallstones (n = 46, 56.8%), benign stenosis (n = 29, 35.8%) and malignant stenosis (n = 6, 7.4%). All stents were made of polyethylene and were placed endoscopically. The median duration of stent placement was 65 days (range 5–1095 days). Cholangitis at the time of stent placement was present in 50 (61.7%) patients. A polybacterial biofilm was detected in most stents (n = 73, 90.1%), while single species were found in the remaining eight (9.9%) cases. The most common Gram-negative bacteria in the cited study were *Pseudomonas* spp. (n = 38), *Citrobacter* spp. (n = 23), *Klebsiella* spp. (n = 22), *Serratia* spp. (n = 16), *Escherichia coli* (n = 14), *Aeromonas* spp. (n = 12), *Proteus* spp. (n = 10) and *Enterobacter* spp. (n = 9). Among the Gram-positive bacteria, the most common were *Staphylococcus* spp. (n = 20), *Streptococcus* spp. (n = 13) and *Enterococcus* spp. (n = 13) [16].

In a prospective study that was conducted in Rome between July 2019 and February 2021 in patients requiring urgent biliary stent exchange/removal due to benign biliary stenosis, the mean duration from stent placement was 120 days [71]. A microbiological analysis of bile and stent samples taken from 22 patients was performed. The dominant species isolated in the bile and stent samples were *Lactobacillus* spp. (7.1 and 13.7% in bile and stent samples, respectively), *Enterococcus faecalis* (9.2% and 9.7%), followed by *Escherichia coli* (8.2 and 9.1%), *Klebsiella pneumoniae* (7.7% and 9.1%, respectively) and *Enterococcus faecium* (6.6% and 6.9%). Among the anaerobic Gram-positive bacteria, the most frequently isolated genus was *Clostridium* spp. (5.1% and 5.1%), especially *C. perfringens* (3.1% and 2.9%), while among the Gram-negative anaerobic bacteria, the most common genus was *Bacteroides* spp. (2.0% and 1.1%), with no differences between the individual species. The most commonly isolated yeast species were *Candida* spp. (11.7% and 8.0%), especially *Candida albicans* (8.7% and 7.4%) [71]. The biofilm which formed inside stents was an organized community of microorganisms enclosed in a self-produced exopolysaccharide matrix containing proteins and other polymers, which grew on a solid, synthetic surface, [16,66].

The experiment conducted in Japan aimed to investigate the antibacterial efficacy of polyurethane biliary stents coated with silver compared to polyurethane stents without this coating. Stent obstruction is caused mainly by the deposition of bile sediments, which consist of cholesterol crystals, calcium bilirubinate, calcium palmitate, bacteria and/or fungi, microbiological by-products, proteins, dietary fiber and glycoproteins. Silver ions proved to be a much stronger inhibitor of biofilm formation than many other antibacterial agents, and at lower concentrations. The observation of an almost complete lack of bacterial adhesion on the surfaces of silver-coated biliary stents after a longer period of time indicated the possibility of achieving the long-term patency of polyurethane stents coated with this metal [63].

## 6. Preventing and Combating Bacterial Biofilm

Because of the medical importance of bacterial biofilms, effective methods of preventing their formation and combating them are of great importance in clinical practice.

Bacterial cells in a biofilm are constantly growing and dispersing. These processes are regulated by complex signaling pathways. Therefore, the mass of the biofilm is constantly changing over days and weeks. The inhibition of biofilm formation cannot be achieved solely by preventing the adhesion of cells or proteins, as each bacterial species has its own surface characteristics that regulate its adhesion to a given surface [13].

The preventive action is aimed at changing the physical properties of the surface by modifying the self-assembled monolayer (SAM) that inhibits bacterial adhesion, disrupts biofilm formation or promotes its removal, in order to prevent the accumulation of a biofilm layer [72,73]. A self-assembled monolayer is a thin, single-layer film of small molecules that are attached to a surface in a highly ordered way [13]. SAMs are made of small molecules; they usually have a thickness in the range of 1–5 nm depending on the size of the molecule, and thus belong to the category of nanoscale materials. Compared to polymer films or metals, SAMs are resistant to release into the surrounding environment due to their strong interaction with the surface [74]. In addition to inhibiting bacterial biofilm formation, the ultimate functionality of SAMs can also be used to retain a biocidal agent on their surface. Most bactericidal SAMs kill by contact, using biocidal agents that act on the outside of the bacteria. In the case of these surfaces, bacteria are killed upon contact [13]. The covalent attachment between the surface and the bactericide is particularly important because it prevents its release, which could lead to the development of bacterial resistance. It also allows the use of relatively low concentrations of the active substance compared to the doses administered in vivo. If the bactericide requires internalization into the bacterial cell to act effectively, it can be used in an SAM, but then the mechanism of action is killing by release, meaning that the SAM releases the bactericide over time. In this case, there may be potential problems with controlling the concentration of the bactericide. This can lead to toxic effects if too much is released at the beginning of the application, and to inactivity when the coating loses its bactericide [13]. To inhibit biofilm formation, SAMs can form quaternary ammonium compounds on gold, titanium or silicone surfaces [75,76]. Another effective strategy for inhibiting the formation of biofilms of Gram-positive *S. aureus* and *S. epidermidis* bacteria is the covalent placement of the drug (vancomycin) on the surface of titanium and stainless steel alloys. Much lower overall concentrations of the antibiotic are used than the therapeutic, while maintaining the antibiotic at the biomedical implant site. As studies have shown, this effect can be maintained even after exposure to serum proteins [13,77]. A study was conducted to investigate the properties of an aminosilanized titanium surface onto which the antiseptic chlorhexidine was grafted using glutaraldehyde as a linker. The resulting surface inhibited the formation of an *S. aureus* biofilm in proportion to the concentration of chlorhexidine used [78]. Similarly, the applied salicylic acid was released into the substrate and showed an up to 90% inhibitory effect on the viability and growth of settled bacteria: *E. coli*, *S. aureus* and *S. epidermidis*. This solution may be suitable for implementation in situations with a limited exposure window, e.g., during the healing period after surgery, although these functionalized surfaces retain their antibiotic activity for a limited time [79]. Metal cations, particularly silver ions, which are known for their antibacterial properties, have also been grafted onto self-assembled monolayers on various surfaces to disrupt the biofilm formation process, in addition to antibiotics, which have also been grafted onto SAMs [80]. Silver has a broad spectrum of antimicrobial activity and, if used in small amounts due to toxicity concerns and so as to minimize costs, can be an effective bactericide [81,82]. Silver cations have been coordinated to titanium and stainless steel surfaces. Studies have confirmed its biocidal efficacy against *E. coli*, *S. aureus*, *S. epidermidis* and *P. aeruginosa*. A silver-coordinating SAM inhibited bacterial cell adhesion by three orders of magnitude and reduced the likelihood of biofilm formation by 80%. This SAM coating has been proven to kill bacteria and prevent their adhesion. The amount of silver required for this effect is less than 1 nmol/cm^2^, which is less than many other antibacterial silver treatments [83]. Copper (cations of Cu^2+^) also has antibacterial properties; its bactericidal effect has been assessed against *E. coli* and *S. aureus*. After five hours, almost 95% of bacterial cells were killed, and more than 99.9% were killed after 24 h [84]. Currently, the chemical composition of the biofilm matrix is known for most pathogenic microorganisms, so it would be realistic to disperse bacterial cells enclosed in biofilms by degrading the matrix. One of the main components of many bacterial biofilms is eDNA. Bacteria produce their own nucleases to digest eDNA, among other things, in order to disperse the biofilm matrix depending on the environmental conditions [85]. eDNA is a polymeric component of the matrix of many bacterial biofilms and most likely originates from cell lysis [86]. Nucleases can therefore become therapeutic agents by destroying the protective matrix and making bacteria sensitive to other treatments [11]. Many *Enterobacterales* produce extracellular amyloid fibrils, which are harmful because of their ability to adhere to surfaces and form and maintain biofilms. Specific bioactive compounds that inhibit the formation of these fibrils have been identified, effectively preventing biofilm formation and destabilizing the mature biofilms of pathogenic *E. coli* [87].

Stent obstruction is a serious problem in the treatment of biliary tract strictures; therefore, some modifications (i.e., design changes, special coatings and new biomaterials) have been proposed to prolong patency time, but there are no definitive data to support the introduction of these solutions into clinical practice [71].

## 7. Future Perspectives

The problem of the internal stent occlusion and the exact cause of its occurrence is not completely solved. The results of various studies have shown that the composition of the sediments from the biliary stent is not particularly dependent on the material from which it is made, the extraction procedure or any of the patient characteristics taken into account [68,88]. The main solution to this problem would be to develop innovative stents made of materials that have permanent antimicrobial properties, thus offering a promising solution to this long-standing problem [71]. Technical developments remain desirable to develop new stent materials and designs that minimize or eliminate the obstruction phenomenon [70]. The implementation of such improvements in stent design could significantly improve patient outcomes and reduce the risk of related complications [71]. Microscopic biofilms can cause serious infections and patient complications, especially in cases involving long-term medical devices. They can also be difficult or impossible to detect without removing the medical device. Further studies should focus on developing surfaces that prevent biofilm formation and facilitate their detection to provide clinicians with additional information and to enable the early identification of potential complications and the sources of these complications [89].

After analyzing the literature, the following main strategies for combating biofilms were identified:

- Development and creation of antiadhesive materials and substances with prolonged properties.
- Inhibition of the attachment of microorganisms to the substrate by using special compounds, and the destruction of biofilms early in their formation.
- Use of compounds that disrupt QS, causing the detachment of biofilms and the destruction of their vital activity.
- Use of physical destruction means (lasers, cold plasma, etc.).
- Development of drugs that destroy the biofilm matrix, facilitating cell access.
- Genetic engineering of phages.
- Use of antibacterials together with matrix-destroying factors.
- Drug-eluting biodegradable biliary stents. The drug administered in this way acts on a specific site, limiting the undesirable effects on the rest of the body, and the speed of its release can be controlled.
- Development of a new stent design with anti-reflux action [15,63,64,90,91,92].

The limitations in the above manuscript result from the small number of studies on this topic, and the fact that the published results are not methodologically consistent, which makes their comparison very difficult. Due to the small number of available data, the authors of this article describe the composition of biofilms to a negligible extent, focusing mainly on the types of isolated microorganisms. In the future, it would be reasonable to relate the pathogens found in biofilms to the duration the drain stays in the patient’s body, the chemical composition of the biofilm and the medical indications for the insertion of a prosthesis.

## Figures and Tables

**Table 1:** Most frequently isolated bacterial species on the surface of implanted medical devices.

Permanent Medical Device for Long-Term Use	Most Frequently Isolated Bacteria
Orthopedic implants [38,50,51,52]	*K. pneumoniae*
	*A. baumannii*
	*S. epidermidis*
	*S. aureus*
Stents [53]	*E. coli*
	*Enterobacter* spp.
	*Klebsiella* spp.
	*P. aeruginosa*
	*E. faecalis*
	*Streptococcus* spp.
	*S. aureus*
	*S. epidermidis*
Cochlear implants [54]	*P. aeruginosa*
	*S. pyogenes*
	*S. epidermidis*
	*S. aureus*
Breast implants [55]	*E. coli*
	*Mycobacterium* spp.
	*S. epidermidis*
	*S. aureus*
	*Streptococcus* spp.
	*Bacillus* spp.
**Medical Device For Short-Term Use**	**Most Frequently Isolated Bacteria**
Urinary catheter [56,57]	*E. coli*
	*P. aeruginosa*
	*K. pneumoniae*
	*A. baumannii*
	*Enterobacter* spp.
	*S. epidermidis*
	*E. faecalis*
Central line catheter [58]	*P. aeruginosa*
	*K. pneumoniae*
	*S. epidermidis*
	*S. aureus*
	*E. faecalis*
Endotracheal tube [59]	*P. aeruginosa*
	*K. pneumoniae*
	*Acinetobacter* spp.
	*Enterobacter* spp.
	*S. aureus*
	*E. faecalis*
Feeding tube [60]	*P. aeruginosa*
	*Enterococcus* spp.
	*Bacillus* spp.
	*Staphylococcus* spp.
Contact lenses [38,56,61]	*E. coli*
	*P. aeruginosa*
	*S. aureus*
